# Risk and resource factors of antisocial behaviour in children and adolescents: results of the longitudinal BELLA study

**DOI:** 10.1186/s13034-021-00412-3

**Published:** 2021-10-22

**Authors:** Christiane Otto, Anne Kaman, Michael Erhart, Claus Barkmann, Fionna Klasen, Robert Schlack, Ulrike Ravens-Sieberer

**Affiliations:** 1 Department of Child and Adolescent Psychiatry, Psychotherapy, and Psychosomatics, University Medical Center Hamburg-Eppendorf, Martinistr. 52, Building W29, D-20246 Hamburg, Germany; 2 Alice Salomon University of Applied Sciences, Berlin, Germany; 3 Apollon University of Applied Sciences, Bremen, Germany; 4 Department of Epidemiology and Health Monitoring, Robert Koch-Institute, General-Pape- Straße 62-66, D-12101 Berlin, Germany

**Keywords:** Antisocial behaviour, Risk and protective factors, Resources, Children and adolescents, Longitudinal BELLA study

## Abstract

**Background:**

Antisocial behaviour is a common phenomenon in childhood and adolescence. Information on psychosocial risk and resource factors for antisocial behaviour are important for planning targeted prevention and early intervention programs. The current study explores risk and resource factors of antisocial behaviour in children and adolescents based on population-based longitudinal data.

**Methods:**

We analysed longitudinal data from the German BELLA study (*n* = 1145; 11 to 17 year-olds) measured at three measurement points covering two years. Latent growth analysis, linear regression models and structural equation modelling were used to explore cross-sectional and longitudinal data.

**Results:**

Based on baseline data, we found that stronger self-efficacy and worse family climate were each related to stronger antisocial behaviour. Longitudinal data revealed that more severe parental mental health problems, worse family climate at baseline, deteriorating family climate over time, and more social support were each associated with increasing antisocial behaviour over time. We further found a moderating effect for family climate.

**Conclusions:**

Our study provides important exploratory results on psychosocial risk, resource and protective factors in the context of antisocial behaviour in children and adolescents, which need confirmation by future research. Our exploratory results point in the direction that family-based interventions for antisocial behavior in children and adolescents may benefit from considering the family climate.

**Supplementary Information:**

The online version contains supplementary material available at 10.1186/s13034-021-00412-3.

## Introduction

Antisocial behaviour is a key symptom and subtype of conduct disorder (CD) as defined by DSM-5 and ICD-10. Antisocial behaviour in children and adolescents can be characterized by symptoms such as being verbally and physically harmful to other people, violating social expectations, engaging in behaviours such as delinquency, vandalism, theft, and truancy, or having disturbed interpersonal relationships, whereby antisocial behaviour among young people is very heterogeneous [1, 2].

Antisocial behaviour and associated conduct disorder are among the most common behavioural problems in childhood and adolescence. According to a systematic review of the global epidemiology of conduct disorder, gender-specific prevalence rates world-wide are relatively stable over time indicating that among 5 to 19 year-olds 3.6% (3.3% to 4.0%) males and 1.5% (1.4% to 1.7%) females are affected [3]. That is, boys are more than two times more likely to be diagnosed with conduct disorder, characterised by heterogeneous patterns of antisocial, aggressive or defiant behaviours, than girls. Symptoms of antisocial behaviour and associated conduct disorder often emerge during preschool years and are most prevalent during middle childhood and adolescence. While antisocial behaviour in childhood is often characterised by milder symptoms such as lying, stealing at home and truancy, more severe symptoms such as aggressive and delinquent behaviour increase during adolescence [4]. In approximately 50 % to 85 % of children and adolescents with an early onset conduct disorder, serious behaviour problems persist into adulthood [5]. A childhood conduct disorder characterised by antisocial behaviour can be a premorbid condition for a antisocial personality disorder in adulthood [2].

Patterson et al. [6] assume that the etiology and the course of antisocial behaviour from childhood through adolescence are results of a multifactorial process. According to Dishion and Patterson [4], relationship dynamics, behaviour settings, self-regulation, and the cultural and community context are the main domains involved in the development of antisocial behaviour. On the other hand, recent research has shown that genetic and environmental influences are also of great importance in this context [7, 8].

Antisocial behaviour and associated conduct disorder often co-occur with other mental disorders, most commonly with attention-deficit/hyperactivity disorder (ADHD), oppositional defiant disorder (ODD), depression and anxiety disorder [5, 9–11]. Furthermore, antisocial behaviour is associated with significant impairments as well as adverse consequences and health outcomes in adulthood such as low educational achievement and unemployment [12], alcohol and drug dependence [13, 14], criminality [15], teenage pregnancy [13] and psychosocial malfunctioning [16, 17]. Moreover, adolescent conduct disorder has a significant impact on the subjective overall health [13]. Taken together, children who exhibit antisocial behaviour are not only affected by impairments in various life domains, but may also cause significant distress in others, which emphasises the high public health relevance of this behavioural problem.

In order to prevent the adverse consequences and impairments associated with antisocial behaviour, targeted prevention and early intervention strategies for children and adolescents under risk are required. Understanding the factors related to the development of antisocial behaviour can help to inform experts planning prevention and intervention. According to the literature, these factors are usually classified into personal, familial, and social risk and resource factors. Risk factors increase the probability of a negative mental health outcome, whereas resource factors support a positive development. Factors that strengthen the mental health of children when being exposed to risks are defined as protective factors [18–21].

While psychosocial risk factors for antisocial behaviour are comparably well studied, studies on resource factors are still rare. Most previous research has focused on the role of familial risk factors. Several cross-sectional and longitudinal studies have found family conflicts [22], coercive or hostile parenting [23, 24], inconsistent discipline and poor supervision [25], parental strain [24], as well as unhealthy family functioning [26] to be associated with antisocial behaviour in children and adolescents. Further, living in disrupted families, meaning that the child is permanently or temporarily separated from a biological parent, may contribute to the development of antisocial behaviour [24]. On the other hand, positive parenting behaviours that are characterised by involvement, support and guidance have been related to prosocial behaviours through anger regulation in adolescents [25]. Previous studies have further revealed that a family history of mental health problems [22, 24] as well as a parental chronic disease [27] are associated with the development of childhood antisocial behaviour and delinquency.

Furthermore, cross-sectional and longitudinal studies found protective effects of self-efficacy on antisocial behaviour in children and adolescents [28, 29]. Self-efficacy is a concept that describes the perception of one’s own ability to achieve goals [30]. Research findings indicated that children and adolescents’ perceptions of self-efficacy for resisting peer pressure are associated with a reduction of antisocial behaviour [28, 29].

Concerning social factors related to antisocial behaviour, Farrington [24] has identified the interaction with antisocial peers as important risk factor, while prosocial involvement and having good social skills may support children’s prosocial behaviour [22]. Moreover, studies have found associations between antisocial behaviour and academic failure as well as low educational achievement, although the direction of these associations is not clear [22, 24]. In terms of socioeconomic factors, cross-sectional studies have demonstrated associations of low family income, low parental education and poor housing with antisocial behaviour in children and adolescents [24, 26, 31]. Further, there is evidence of an association between migration background and more pronounced antisocial behaviour [32].

Overall, a number of previous studies focused on specific risk, resource and/or protective factors associated with antisocial behaviour. However, most of these studies analysed only cross-sectional data. Moreover, in most studies only direct associations of risk and resource factors with antisocial behaviour have been examined, although research has shown that risk and resource factors can interact in different ways [33].

The present study explores cross-sectional and longitudinal effects of the risk factor parental mental health problems and individual, familial, and social resource factors on the state and change in antisocial behaviour in children and adolescents over time based on data of a German population-based sample. We expected that parental mental health problems (risk factor) and self-efficacy, family climate, and social support (resource factors) are not only associated with initial antisocial behaviour, but also with the change in antisocial behaviour over time. Following the approach proposed by Masten [18], we expected that resource factors might have the potential to act as protective factors. Therefore, we further explored whether the examined resource factors act as protective factors moderating the association between the risk factor parental mental health problems and antisocial behaviour initially and over time.

Based on the literature we had the following expectations concerning cross-sectional data:

- Stronger parental mental health problems (risk factor) are associated with stronger antisocial behaviour in children and adolescents.
- Higher self-efficacy, better family climate and more social support (resource factors) are each associated with less antisocial behaviour.
- Children and adolescents who are not living with both biological parents, whose parents have a chronic disease, and whose parents report more severe strain show stronger antisocial behaviour.
- Boys, older children and adolescents, those with a lower SES, and children and adolescents with a migration background show stronger antisocial behaviour.

We had the following expectations concerning our analyses of longitudinal data:

- Increasing parental mental health problems (risk factor) are associated with increasing antisocial behaviour in children and adolescents over time.
- Increasing self-efficacy, family climate and social support (resource factors) are each associated with decreasing antisocial behaviour over time.

Further, we had the following expectations concerning interactions between risk and resource factors:

- High self-efficacy, a good family climate and good social support (resource factors) each attenuate the detrimental effect of parental mental health problems (risk factor) on antisocial behaviour.
- Increasing self-efficacy, family climate and social support (resource factors) each attenuate the detriemental effect of increasing parental mental health problems (risk factor) on the change in antisocial behaviour over time.

## Methods

### Study

We analyzed data from the longitudinal BELLA study [34]. The BELLA study is the mental health module of the German National Health Interview and Examination Survey for Children and Adolescents (KiGGS) [35]. In the BELLA study data on mental health, health-related quality of life, and associated risks and resources in German children, adolescents, and young adults have been collected. The KiGGS and BELLA study are conducted in close cooperation; baseline assessments of both studies took place from 2003 to 2006. From the KiGGS baseline sample (*n* = 17,641 children and adolescents aged 0 to 17 years) a subsample was drawn for the BELLA study by random (*n* = 2942 children and adolescents, 7 to 17 years old). The team of the BELLA study informed these children and adolescents and their parents about the study and asked for their participation. The final BELLA baseline sample included *n* = 2863 (response rate: 97.3%) children and adolescents (aged 7 to 17 years) and their parents. Further measurement points of the BELLA study were conducted with *n* = 2423 of the BELLA baseline participants (84.6%) taking part in the 1-year follow-up (2004 to 2007) and *n* = 2190 (76.5%) of the baseline participants taking part in the 2-year follow-up (2005 to 2008). Data for the BELLA study was gathered by means of computer-assisted telephone interviews and subsequent paper-pencil questionnaires. Trained interviewers conducted the telephone interviews following structured guidelines, regular supervisions were provided by a child and adolescent psychologist. Participants received a small incentive in the form of a 5 Euro gift card. Self-reported data was collected from participants aged at least 11 years, and parent-reported data from one parent of each participant. Standardised, psychometrically sound and internationally tested measures were administered (if available). The ethics committee of the University Hospital Charité in Berlin and the Federal Commissioner for Data Protection in Germany both gave their approvals for the BELLA study. More details on the design and methods of the longitudinal BELLA study are published providing detailed information on the sampling of the BELLA study (which results from the larger KiGGS survey) [36] and drop out analyses for BELLA follow-up assessment; already reported drop out analyses indicated that participants with lower SES or migration background were more often lost to follow-up assessments, but gender, community size, region (Eastern vs. Western Germany), parent-reported general health or parent-reported mental health scores at baseline were not related to drop-out status [34].

### Participants

We analysed data from the first three measurement points of the BELLA study (baseline, 1-year and 2-year follow-ups) in the present study. We could include cases in our analyses, if (i) data gathered only at baseline were completely available (on age, gender, socioeconomic status (SES), migration status, single parent family, step parent, parental chronic disease, and parental strain) and if (ii) longitudinally measured data were available for at least one measurement point (on antisocial behaviour and comorbid symptoms of depression, generalised anxiety, and ADHD, on parental mental health problems, self-efficacy, family climate, and social support). Further, cases were only analysed if the same parent had fulfilled the parent questionnaire at each measurement point. The final sample consisted of *n* = 1145 children and adolescents aged 11 to 17 years at baseline.

### Measures

#### Sociodemographic variables

We determined age (in years), gender, the SES and migration status at baseline. The SES was measured in the KiGGS study with the parent-reported Winkler-index [37] which gathers information on education, profession and income of both parents. We used the sum-score of the Winkler-index (range: 3 to 21, with higher values indicating better SES) in the following analyses. For sample description only, we categorised the sum-score to differ between participants with low (scores from 3 to 8), middle (scores from 9 to 14) and high SES (scores from 15 to 21) [38]. Migration status was determined in the KiGGS study according to Schenk [39], if (i) the child or adolescent had immigrated to Germany and had at least one parent born in a country other than Germany, or if (ii) both parents immigrated to Germany or did not hold German citizenship.

#### Familial and parental risks

At baseline the family structure was assessed in the KiGGS study asking the parents with whom the participating child or adolescent lived at home (response options = with both biological parents, with the mother (and her partner), with the father (and his partner), with grandparents or other relatives, with step- or foster parents or in a children’s home). For the following analyses, we recoded this variable to identify children and adolescents who did not live with both biological parents (code: 1) versus those who did (code: 0). In the baseline assessment of the BELLA study, parents were asked for chronic diseases/disabilities and fulfilled a short questionnaire on parental strain. We gathered the responses to both items on chronic diseases/disabilities (“Do you have a chronic disease (e.g., asthma, diabetes, rheumatism) or disability?” and “Does your partner have a chronic disease (e.g., asthma, diabetes, rheumatism) or disability?”) and a created a new variable indicating whether at least one parent of the child or adolescent had a chronic disease or disability (code: 1), or whether no parent was affected (code: 0). To measure parental strain, we used 11 items asking for the particular burden caused e.g., by housekeeping, financial problems, job-related issues, being a single parent, or by caring for an ill family member [19]. Parents rated the perceived strain for each burden by means of a 5-point response scale (0 = “none” to 4 = “very strong”). For our analyses, we calculated a mean over all items with a higher score indicating more severe parental strain.

#### Antisocial behaviour

We assessed antisocial behaviour in children and adolescents by parent-reports at each measurement point based on the German version of the well-established Child Behavior Checklist (CBCL) [40, 41]. The CBCL offers a subscale on delinquency including 13 items (“Behavior of your child:”, e.g., “Steals at home”, “Lying or cheating”), each offered with three response options (0 = “not true” to 2 = “very true or often true”). We calculated the mean across the items with a higher mean indicating more severe antisocial behaviour. Acceptable internal consistency was found for this scale in the sample under analysis (Cronbach’s α ranged from 0.70 to 0.73 across measurement points).

#### Comorbid symptoms of attention deficit hyperactivity disorder (ADHD), depression, and generalised anxiety

We measured comorbid symptoms in children and adolescents longitudinally at each investigated measurement point. We recoded items if necessary and calculated means for each symptom scale with higher values indicating stronger symptoms.

Parent-reported symptoms of attention deficit hyperactivity disorder (ADHD) were assessed based on the Conners Global Index (C-GI) [42, 43]. In the BELLA study, a German version of the C-GI was developed and administered [44–46]. For the present analyses, the C-GI subscale restless-impulsivity was used including overall seven items on inattention (e.g., “inattentive, easily distracted”), hyperactivity (“fidgeting”) and impulsivity (“excitable, impulsive”). Each item was offered with a 4-point response scale (0 = “not true at all” to 3 = ”very much true”). Acceptable to good internal consistency was found for the C-GI scale restless-impulsivity in the investigated sample (α ranged from 0.77 to 0.81).

Self-reported depressive symptoms were assessed by means of the German version of the established Center for Epidemiologic Studies Depression Scale (CES-DC) [47, 48]. This measure gathers emotional, cognitive and behavioural aspects of depression (e.g., “I thought my life had been a failure”) with overall 20 items, each presented with a 4-point response scale (0 = “not at all” to 3 = “a lot”). Good internal consistency was given for the CES-DC in our sample (α ranged from 0.81 to 0.87).

Self-reported symptoms of generalised anxiety were measured based on a German version of the Screen for Child Anxiety Related Disorders (SCARED-D) [49–51]. The scale on generalised anxiety of the SCARED-D includes 9 items (e.g., “I worry about being as good as other kids”) offered with a 3-point response scale (0 = “not true or hardly ever true” to 2 = “very true or often true”). The internal consistency for this scale was good in our sample (α ranged from 0.81 to 0.85).

#### Risk and resource factors

We measured risk and resource factors longitudinally, at each measurement point. Items were recoded if necessary, and scores across the items of each scale were calculated. We calculated means for scale scores with a higher mean indicating more pronounced self-efficacy, better family climate, better social support or stronger parental mental health problems, respectively.

The risk factor parental mental health problems was measured by parent-reports using the Symptom-Check List 9-item Short version (SCL-S-9) [52], which is a short version of the SCL-90-R [53]. The SCL-S-9 serves to assess a wide range of psychopathologic symptoms with each item belonging to one dimension of the original SCL-90-R (i.e., somatization, obsessive-compulsive, interpersonal sensitivity, depression, anxiety, hostility, phobic anxiety, paranoid ideation, and psychoticism). Each of the nine items of the measure are presented with a 5-point response scale (0 = “none at all” to 4 = “very severe”). Good internal consistency was found for the SCL-S-9 in the investigated sample (α was 0.81 at each measurement point).

The individual resource factor self-efficacy in children and adolescents was measured by self-reports using the General Self-Efficacy Scale (GSE) [54, 55]. The GSE includes 10 items (e.g., “If I am in trouble, I can usually think of a solution”) provided with a 4-point response scale each (0 = “not at all true” to 3 = “exactly true”). The internal consistency was good for the GSE in our sample (α ranged from 0.81 to 0.83).

The familial resource factor family climate was measured by self-reports based on the German Family Climate Scale (FCS) [56]. The FCS represents the German adaptation of the Family Environment Scale (FES) [57]. We administered eight items of the FCS in the BELLA study which are related to active recreational organization and cohesion (e.g., “In our family everybody cares about each other’s worries”) and presented with a 4-point response scale each (0 = “not true” to 3 = “exactly true”). Acceptable to good internal consistency was given for the administered FCS in the investigated sample (α ranged from 0.78 to 0.82).

The social resource factor social support was assessed via self-reports gathered from children and adolescents. For administration in the BELLA study, eight selected and translated items from the Medical Outcomes Study Social Support Survey (SSS) [58] were administered. The administered items measure how frequent specific types of support are available (“How often is the following type of support available for you if you need it?” e.g., “Someone who listens”) and are provided with a 5-point response scale each (0 = “none of the time” to 4 = “all of the time”). The internal consistency was good to excellent for this short version (SSS-short) in our sample (α ranged from 0.88 to 0.91).

### Data analysis

Latent growth modelling is often used to investigate changes in behaviours [59]. By means of a latent growth model (LGM), two latent parameters are estimated with the intercept representing the initial state of a variable under analysis at baseline and the slope reflecting the change in this variable over time. In the present study, we used this approach and followed a two-step analysing procedure. We started by calculating a LGM for each construct which was longitudinally measured (i.e., antisocial behaviour, the investigated risk and resource factors, and symptoms of comorbid disorders). Goodness of fit was assessed via the root mean square error of approximation (RMSEA) and the comparative fit index (CFI) for each LGM. We then used intercepts and slopes resulting from LGMs in linear regression models. By means of regression Model A0, we explored effects of the initially measured risk and resource factors on initial antisocial behaviour. By means of Regression Model B0, we explored effects of initially measured risk and resource factors as well as effects of the changes in these constructs over time on the change in antisocial behaviour over time. In each of these models we considered the following covariates: sociodemographic information (i.e., age, gender, SES, and migration status), data on familial and parental risks (i.e., living with at least one non-biological parent, parental chronic disease, and parental strain), and data on comorbid symptoms (of depression, generalised anxiety, and ADHD). In order to explore associations between the considered constructs and antisocial behaviour age-group specifically, we re-run both regression models separately for 11 to 13 year-olds and for 14 to 17 year-olds.

By means of two further regression models, we explored whether the investigated resource factors (i.e., self-efficacy, family climate and social support) serve as protective factors in terms of moderating the relationship between the risk factor parental mental health and antisocial behaviour in children and adolescents. Regression Model A1 was conducted based on baseline data adding interaction effects between parental mental health problems and each resource factor to Model A0. Model B1 was conducted adding all potential interaction effects between parental mental health problems and each resource factor to Model B0 using longitudinal data.

In each regression model, we used centered metric variables. Further, we interpreted standardised regression coefficients as correlation coefficients to allow rough interpretation of the strengths of detected associations (*r* = 0.10 indicates a weak, *r* = 0.30 a medium and *r* = 0.50 a strong association).

We additionally calculated a structural equation model (SEM) focusing on the exploration of associations between longitudinally measured risk and protective factors, and antisocial behavior in order to evaluate the results found by means of regression Models A0 and B0. In the SEM, we specified direct paths from the intercepts of the risk and resource factors on the intercept and on the slope of antisocial behavior; direct paths from the slopes of the risk and ressources on the slope of antisocial behaviour were also specified and estimated according to the maximum likelihood criterion. The latent parameters of the risk and resource factors as well as the intercept and the slope of antisocial behaviour were freed to correlate. In line with the LGMs, we fixed time scores 0, 1 and 2 for the estimation of the slopes to reflect equidistant measurement points (with 0 representing the baseline assessment) [60], and we determined model fit.

Mplus 8 [60] was used for LGMs and for the SEM, IBM SPSS 26 for regression models.

## Results

The analysed sample included *n* = 1145 children and adolescents aged 11 to 17 years at baseline (Table 1). In this sample, about half of the children and adolescents were female, the mean age was about 14 years, about half of the participants lived in families with a medium SES (low SES: 19%, *n* = 219; medium SES: 51%, *n* = 587; high SES: 30%, *n* = 339), and 6% (*n* = 71) of the children and adolescents had a migration background (*n* = 37 were born in other countries, i.e., Russia (*n* = 8), Kazakhstan (*n* = 5), Bosnia and Herzegovina, Greece, Romania, and Ukraine (*n* = 2 each), and Austria, Belarus, Brazil, Cuba, Czech Republic, Egypt, Finland, Kossovo, Luxembourg, Moldova, Paraguay, Poland, the Netherlands, the US, Turkey, and Uzbekistan (*n* = 1 each); *n* = 34 were born in Germany with mother and/or father born in Poland and Russia (*n* = 8 each), Turkey (*n* = 7), Romania (*n* = 3), Austria, China, Croatia, Egypt, France, Greece, India, Kossovo, Peru, the United Kingdom, and Serbia and Montenegro (*n* = 1 each)). For each participant, the same parent completed the parent questionnaire in the BELLA study gathering information on parent-reported antisocial behaviour and ADHD in children and adolescents, on parental mental health problems, and parental strain. For 91% of the participants the mothers (*n* = 1045), for 8% the fathers (*n* = 87), and for 1% step-, foster- or grandparents (*n* = 13) completed the parent questionnaire.

**Table 1:** Sample description of children and adolescents aged 11 to 17 years (at baseline)

	Baseline		1-year follow-up		2-year follow-up	
	n (%)	M (SD)	n	M (SD)	n	M (SD)
Sociodemographic data ^1^						
Female	583 [51]					
Age (in years)		13.83 (1.96)				
Socioeconomic status (possible range: 3–21)		12.27 (4.10)				
Migration background	71 [6]					
Familial and parental risks						
Not living with both biological parents	163 [14]					
Parental chronic disease (at least one parent)	363 [32]					
Parental strain (possible range: 0–4)		0.81 (0.60)				
Antisocial behaviour (possible range: 0–2)	1138	0.13 (0.18)	903	0.13 (0.17)	786	0.13 (0.17)
Comorbid mental health problems						
Symptoms of ADHD (possible range: 0–3)	1145	0.66 (0.50)	902	0.58 (0.49)	894	0.54 (0.45)
Depressive symptoms (possible range: 0–3)	1128	0.48 (0.33)	876	0.45 (0.32)	852	0.44 (0.35)
Symptoms of generalised anxiety (possible range: 0–2)	1128	0.63 (0.37)	876	0.60 (0.39)	852	0.59 (0.40)
Risk factor						
Parental mental health problems (possible range: 0–4)	1145	0.56 (0.49)	902	0.55 (0.49)	894	0.47 (0.44)
Resource factors						
Self-efficacy (possible range: 0–3)	1128	2.14 (0.38)	875	2.16 (0.42)	852	2.18 (0.39)
Family climate (possible range: 0–3)	1135	1.83 (0.53)	888	1.84 (0.52)	761	1.81 (0.52)
Social support (possible range: 0–4)	1130	3.11 (0.74)	890	3.29 (0.66)	760	3.33 (0.64)

^1^Sociodemographic information and data on familial and parental risks were available for the complete sample under analysis (*n =* 1145)

*ADHD* attention-deficit/hyperactivity disorder, *M* mean, *SD* standard deviation; for measures see text (Methods)

Results for Model A0 using cross-sectional baseline data are depicted in Table 2. Findings indicated that stronger antisocial behaviour was related to older age and lower SES. Further, antisocial behaviour was more likely in children and adolescents who did not live with both biological parents and was associated with more severe parental strain. Moreover, stronger antisocial behaviour was related to more severe comorbid symptoms of ADHD, stronger depressive symptoms, and less symptoms of generalised anxiety. No effect was found for the risk factor parental mental health problems, but we found significant effects for two of the three investigated resource factors. More pronounced antisocial behaviour at baseline was associated with stronger self-efficacy and worse family climate. Detected effects indicated negligible to small associations of not living with both biological parents, comorbid symptoms of generalised anxiety and both resource factors with antisocial behaviour; we found small associations of age, SES, parental strain, and comorbid depressive symptoms, and a medium association of comorbid symptoms of ADHD with antisocial behaviour at baseline.

We added results of Model B0 using longitudinal data to Table 2. Increasing antisocial behaviour was related to younger age, less parental strain (both at baseline), and increasing comorbid symptoms of ADHD over time. Increasing antisocial behaviour over time was further associated with more severe parental mental health problems (risk factor) at baseline, worse family climate (resource factor) at baseline, deteriorating family climate over time, and with more social support (resource factor) at baseline. Detected effects indicated a negligible to small association of initial parental mental health problems with the change in antisocial behaviour, and small associations for all remaining effects.

Please note, the fit was good for most LGMs according to the RMSEA and the CFI using guidelines for interpretation from Schermelleh-Engel et al. [61] (antisocial behaviour: χ² = 0.201, degrees of freedom (df) = 1, RMSEA= 0.000 (90 % Confidence Interval (CI): 0.000–0.060), CFI= 1.00; ADHD: χ² = 3.020, df = 1, RMSEA= 0.042 (CI: 0.000–0.100), CFI= 1.00; depressive symptoms: χ² = 0.025, df = 1, RMSEA= 0.000 (CI: 0.000–0.040), CFI= 1.00; generalised anxiety: χ² = 0.304, df = 1, RMSEA= 0.000 (CI: 0.000–0.064), CFI= 1.00; self-efficacy: χ² = 0.354, df = 1, RMSEA= 0.000 (CI: 0.000–0.065), CFI= 1.00; family climate: χ² = 0.901, df = 1, RMSEA= 0.000 (CI: 0.000–0.077), CFI= 1.00). However, the fit for the LGMs for parental mental health problems (χ² = 8.961, df = 1, RMSEA= 0.083 (CI: 0.040–0.137), CFI= 0.99) and for social support (χ² = 11.013, df = 1, RMSEA= 0.094 (CI: 0.049–0.147), CFI= 0.97) was not acceptable according to the RMSEA, but good in comparison to the baseline model according to the CFI. Correlations between intercepts and slopes were positive and small for generalized anxiety (*r* =.10, *p* = .001) and depressive symptoms (*r =* 0.23, *p* < 0.001), negative and small for family climate (*r =* − 0.21, *p* < 0.001) and self-efficacy (*r =* − 0.25, *p* < 0.001), negative and moderate for social support (*r =* − 0.31, *p* < 0.001), ADHD (*r =* − 0.36, *p* < 0.001) and antisocial behaviour (*r =* − 0.41, *p* < 0.001), and negative and strong for parental mental health problems (*r =* − 0.63, *p* < .001),

Results of age-group specific models can be found in Additional file 1: Tables S1 and S2). Focusing on the risk factor parental mental health, we found no significant effect at all in our age-group specific analyses. For the investigated protective factors among 11 to 13 year-olds, lower initial social support was associated with more pronounced initial antisocial behaviour; further increasing self-efficacy, lower initial family climate, decreasing family climate, and higher initial as well as increasing social support were each associated with increasing antisocial behaviour over time. In 14 to 17 year-olds, lower initial family climate was associated with more pronounced antisocial behaviour and decreasing family climate was related to increasing antisocial behaviour over time.

Moreover, we calculated interaction models to explore corresponding effects for the resource factors on the association between parental mental health problems (risk factor) and antisocial behaviour. The results are offered in Additional file 1: Table S3. We found no moderating effects for any investigated resource factor based on baseline data (Model A1). However, based on longitudinal data (Model B1) we detected a moderating effect indicating that family climate served as a protective factor; improving family climate over time attenuated the association between increasing parental mental health problems and increasing antisocial behaviour over time; the detected interaction effect indicated a small association (ß = − 0.10; *p* = 0.020).

**Table 2:** Predicting the initial state and change of antisocial behaviour in children and adolescents

	Regression Model A0^a^ predicting initial antisocial behaviour			Regression Model B0^b^ predicting change in antisocial behaviour		
	*b*	β	*p*	*b*	β	*p*
*Constant*	0.13		<0.001	0.00		0.403
Sociodemographic data^c^						
Female	− 0.01	− 0.03	0.319	0.00	0.02	0.528
Age (in years at baseline)	0.01	0.10	0.007	0.00	− 0.12	0.008
Age by gender	0.00	0.02	0.616	0.00	0.01	0.852
Socioeconomic status (at baseline)	0.00	− 0.11	<0.001	0.00	0.03	0.341
Migration background	0.00	0.00	0.926	0.00	− 0.04	0.188
Familial and parental risks						
Not living with both biological parents	0.04	0.09	<0.001	0.00	0.01	0.657
Parental chronic disease (at least one parent)	0.00	− 0.01	0.872	0.00	− 0.01	0.809
Parental strain	0.06	0.24	<0.001	− 0.01	− 0.21	<0.001
Comorbid mental health problems						
Initial symptoms of ADHD (intercept)	0.14	0.39	<0.001	0.00	0.01	0.878
Change in symptoms of ADHD (slope)				0.05	0.13	<0.001
Initial depressive symptoms (intercept)	0.11	0.14	<0.001	− 0.01	− 0.03	0.396
Change in depressive symptoms (slope)				0.00	0.00	0.939
Initial symptoms of generalised anxiety (intercept)	− 0.04	− 0.07	0.031	0.01	0.05	0.217
Change in symptoms of generalised anxiety (slope)				− 0.01	− 0.02	0.527
Risk factor						
Initial parental mental health problems (intercept)	− 0.01	− 0.02	0.571	0.01	0.09	0.042
Change in parental mental health problems (slope)				0.01	0.03	0.457
Resource factors						
Initial self-efficacy (intercept)	0.03	0.06	0.038	0.00	0.00	0.974
Change in self-efficacy (slope)				0.01	0.04	0.261
Initial family climate (intercept)	− 0.03	− 0.09	0.002	− 0.01	− 0.11	0.003
Change in family climate (slope)				− 0.03	− 0.11	< 0.001
Initial social support (intercept)	− 0.01	− 0.02	0.475	0.01	0.11	0.005
Change in social support (slope)				0.01	0.05	0.100

*ADHD* attention-deficit/hyperactivity disorder; *b* unstandardised regression coefficient, *β*  standardised regression coefficient; for measures see text (Methods)

^a^Linear regression Model A0 (*n* = 1145); model fit: adjusted *R*^2^ = 0.35; *F* = 42.38

^b^Linear regression Model B0 (*n* = 1145); model fit: adjusted *R*^2^ = 0.07; *F* = 4.67

^c^We entered all variables simultaneously

**Fig. 1:**
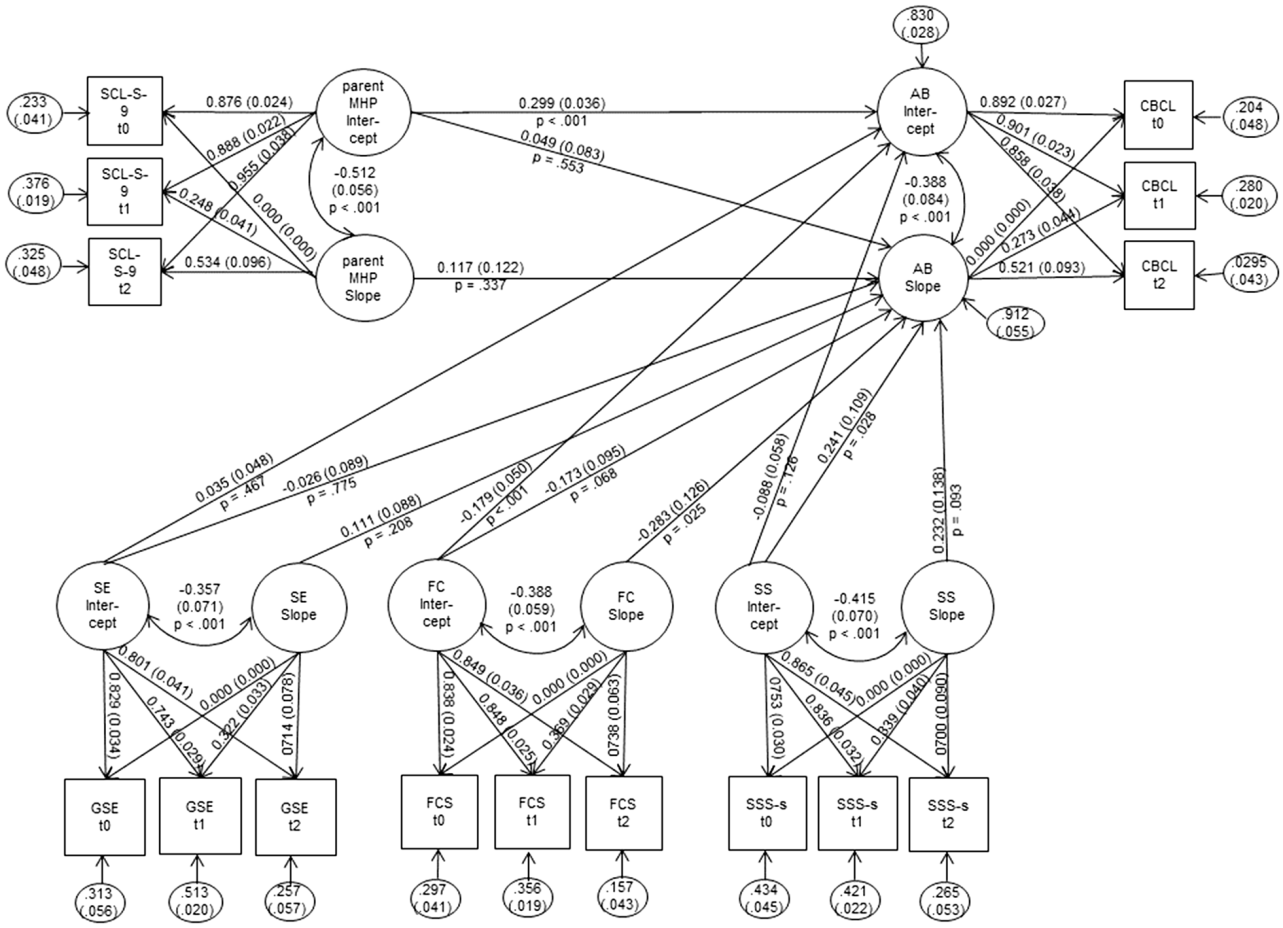
Structural equation model on risk and resource factors of antisocial behaviour in children and adolescents. Standardised estimates (standard errors) are presented, further paths among all intercepts and slopes of risk and resource factors were estimated in the model (not shown for presentation purposes). AB = antisocial behaviour, parent MHP = parental mental health problems, SE = self-efficacy, FC = family climate, SS = social support, CBCL = Delinquency subscale of the Child Behavior Checklist [40, 41]; SCL-S-9 = Symptom-Check List Short version-9 [52]; GSE = General Self-Efficacy Scale [54, 55]; FCS = eight-item score based on the Family Climate Scale [56]; SSS-s = short social support scale with eight items of the German version of the Medical Outcomes Study Social Support Survey [58]

Finally, we specified and calculated the SEM which had a good fit (χ^2^ = 107.79, df = 59, RMSEA = 0.027 (CI = 0.019–0.035), CFI = 0.99). Under the assumption that the specified model represented a correct description of the relationships between observed variables and latent concepts, stronger initial parental mental health problems were associated with more pronounced initial antisocial behaviour (standardised path coefficient = 0.299, *p* < 0.001). Further, good initial familial climate was related to initially less antisocial behaviour (standardised path coefficient = − 0.179, *p* < 0.001). Moreover, improving familial climate was associated with decreasing antisocial behaviour over time (standardized path coefficient = − 0.283, *p* = 0.025). Finally, initial social support was associated with change in antisocial behaviour over time; the standardised path coefficient (0.241, *p* = 0.028) indicated that better social support was related to increasing antisocial behaviour over time. Figure 1 presents results of the SEM.

## Discussion

The aims of the present study were to explore the cross-sectional and longitudinal associations between potential risk and resource factors, and antisocial behaviour in children and adolescents. We used latent growth modeling and linear regression models; additionally we calculated an SEM which provided consistent results based on our longitudinal data. Contrary to our expectation, we found no association between the risk factor parental mental health problems and antisocial behaviour at baseline. However, more severe parental mental health problems at baseline were related to increasing antisocial behaviour over time. Further, we detected associations between the examined resource factors and antisocial behaviour, namely that stronger self-efficacy and worse family climate were related to stronger antisocial behaviour at baseline. Additionally, worse family climate at baseline, deteriorating family climate over time, and more social support at baseline were each associated with increasing antisocial behaviour over time. We further detected a moderating effect for family climate on the relationship between parental mental health problems and antisocial behaviour over time. Moreover, as expected, older age, lower socio-economic status, not living with both biological parents, and more severe parental strain were each associated with stronger antisocial behaviour at baseline, whereas younger age and less parental strain were related to increasing antisocial behaviour over time. Future research is needed to confirm the results of our exploratory study.

In our analyses based on baseline data, the risk factor parental mental health problems did not predict initial antisocial behaviour. This finding is contrary to previous research indicating that parental mental health problems are an important risk factor for antisocial behaviour in children and adolescents [22, 24]. A closer examination of the literature, however, reveals that previous studies mainly focused on specific parental mental health problems such as substance use problems or a family history of antisocial behaviour. The fact that we only investigated parental psychopathology in general, using a short screening questionnaire, may at least partly explain why we were not able to find the expected relationship. It may also be that parents who have mental health problems themselves are less aware of their child’s antisocial behaviour [62, 63]. In our longitudinal model, however, more severe parental mental health problems were associated with increasing antisocial behaviour over time, but we could not confirm this finding in age-group specific analyses. Especially clinical research may contribute further information to evaluate the importance of considering especially parental mental health problems in treatments of child and adolescent antisocial behaviour.

In line with our expectations and previous research on familial influences [22, 26], the resource factor family climate predicted antisocial behaviour in our study initially as well as over time. Children living in families with a worse family climate showed more pronounced antisocial behaviour. Moreover, a deteriorating family climate over time was associated with increasing antisocial behaviour over time. In our moderator model based on longitudinal data, we further found that improving family climate over time attenuated the association between increasing parental mental health problems and increasing antisocial behaviour over time. Therefore, the family climate can be understood as a resource and a protective factor in our study. Future research may confirm our exploratory findings, which seem to point in the direction that children and adolescents with antisocial behaviour may benefit particularly from family-based interventions that address unhealthy family functioning and promote family cohesion and communication. In line with our finding, previous research has shown that family-based interventions and parent training programs are effective in treating children and adolescents with conduct disorder, antisocial behaviour, and delinquency [64, 65].

Moreover, as expected, we found an association between the resource factor self-efficacy and antisocial behaviour at baseline in our study. The direction of this association, however, was not as expected. Children and adolescents with stronger self-efficacy displayed more pronounced antisocial behaviour. Our result, however, is in line with the social-cognitive learning theory [66, 67]. For instance, instrumental-aggressive behaviour (i.e., proactive aggression) can lead to individual success or gain (e.g., to dominance through intimidation of the weaker, material gain through theft, etc.) [68]. Results of the KiGGS baseline study point to the same direction. Youths who had proved to be perpetrators or multiple perpetrators of violence also reported more social support and higher self-efficacy expectations [69]. In this context, affected children may benefit from cognitive-behavioural therapies (CBT) in which they reflect on their behaviour and self-perception. Interestingly, we found only for 11 to 13 year-olds an association between increasing self-efficacy and increasing antisocial behaviour in age-group specific analyses. Future reseach should investigate this association further considering the mixed evidence on the development of self-efficacy in childhood and adolescence [70].

Our finding on the association between social support and antisocial behaviour points in a similar direction. Higher levels of social support were related to increasing antisocial behaviour over time and thus, social support did not appear as a resource factor in our study. These results should be interpreted with care, since the administered items do not explicitly refer to friends or peers. On the other side, our findings may reflect the supportive response of a healthy social environment on antisocial behaviour in children and adolescents. It could further be the case that high levels of support from friends and peers could encourage children in their antisocial actions, especially if the peers also behave antisocial [5, 24]. A corresponding association had previously been observed in a study on violent youth [69]. It remains unclear to what extent the perceived social support we found in our study represents a resource rather than an effect of mutual stabilization through the association of deviant youth [71]. This may be subject to further research, especially since we found associations between social support and antisocial behaviour in age-groups specific analyses only among 11 to 13 year-olds. In this context, social skills training with at-risk children and adolescents may be effective in order to support social competencies and to promote prosocial behaviour. The effectiveness of social skills training for children who are at risk or display antisocial behaviour has been widely researched and proven in several studies [65, 72].

Based on the analysis of baseline data, we further found that older age, lower socio-economic status, not living with both biological parents, and more severe parental strain were each associated with stronger antisocial behaviour, confirming results from previous studies investigating these relationships [4, 24, 26]. These findings underline the need for targeted early prevention and intervention programs in specific vulnerable groups, for example in socially disadvantaged communities. Moreover, we detected that increasing antisocial behaviour over time was related to less parental strain at baseline. Again, it may be that stressed parents are less aware of their child’s antisocial behaviour. In addition, the limited parental strain could possibly be linked to parental neglect, as adverse childhood experiences such as parental abuse and neglect have often been associated with antisocial behaviour in children [73, 74]. Further research is needed to clarify this association. In contradiction to the theory and previous research [3], we found no effect of gender on antisocial behaviour. Future studies could examine whether the association between gender and antisocial behaviour has changed over recent years. Further, we found no effect of migration on antisocial behaviour in our study, which may be related to the fact that only 6% of the participants in our study had a migration background.

In terms of the comorbid mental health problems explored in the current study, we found that stronger antisocial behaviour in children and adolescents was related to more severe symptoms of ADHD and depression, which coincides with results of former research [10, 11]. In our longitudinal model, we further found that increasing antisocial behaviour was related to increasing comorbid symptoms of ADHD. This finding may indicate that the symptoms of antisocial behaviour and ADHD are closely associated, interact and develop concurrently [75, 76]. Contrary to previous research [11], stronger antisocial behaviour was also related to less symptoms of generalised anxiety in our sample. This deviation from previous studies may at least partly be due to the fact that we assessed antisocial behaviour by parent-reports and generalised anxiety by self-reports. However, our finding may not be surprising considering that antisocial behaviour is characterised by criminal and aggressive behaviour, which from a clinical perspective is not a common characteristic of anxiety disorder [6]. Future studies may wish to investigate this association further in greater detail.

This study has some limitations. First and foremost, the present study is only exploratory, future research is needed to confirm our findings and to analyse some above described aspects in more detail. Regarding the presented analyses, it should be further beared in mind that we could not test cause-effect relationships in our study. In order to provide a clear presentation, we considered antisocial behaviour as the successor and the risk and the ressource factors as antecendents while acknowledging that in reality the relationships between these concepts might be more complex and dynamic. Moreover, we could explain 35% of the variance in antisocial behaviour by means of our baseline model, but we could only explain 7% of the variance in the corresponding slope with our longitudinal model. Detected effects only indicate small associations between corresponding variables in our longitudinal model. These findings may reflect that we investigated a general population sample (with rather low levels of mental health problems, and rather good self-efficacy, family climate, and social support). Further, our study only covered a period of two years and the slope for comorbid depressive symptoms did not vary significantly across individuals. Future studies may aim to cover a longer period of time in the lives of children and adolescents. However, these results may as well indicate that the development of antisocial behaviour is associated with important factors that we did not consider in analyses. These factors may include parental substance use [22], genetic and environmental influences [7, 8], personality patterns [77], intelligence [78] as well as neuropsychological correlates [79]. Future studies on risk and resource factors for antisocial behaviour may take these aspects into account. Furthermore, we only differentiated between children and adolescents who lived with both biological parents and those who did not. It must be critically noted that the group of children who did not live with both biological parents is very heterogeneous and included e.g., children who lived with their mothers and their long-term partners, or children who lived in welfare institutions. It is also conceivable that the family status could have influenced the participation rate. These aspects may be investigated in more detail in future studies.

The present study has several strengths. We analysed data of the German BELLA study, which is an important population-based longitudinal study on mental health and well-being of children and adolescents. The large sample size and the wide age range from childhood to young adulthood are considerable strengths. Moreover, we administered established measurement tools to assess the analysed constructs. We used self-reported data of children and adolescents to assess the resource factors as well as comorbid internalizing symptoms of depression and anxiety. Parental psychopathology, antisocial behaviour, and symptoms of ADHD were measured by parent-reports since research has shown that externalizing problems are better observable by parents [80, 81]. We further included familial and parental risks as important covariates in our models. Lastly, using latent growth modelling and linear regression models, we were able to analyse changes in antisocial behaviour as well as changes in risk and resource factors over time.

Overall, the present exploratory study adds to the literature by investigating the longitudinal influences of psychosocial risk and resource factors on antisocial behaviour in children and adolescents. The results point in the direction that parental mental health problems may have detrimental effects on the development of antisocial behaviour. On the other side, a good family climate can have beneficial effects on the state and change in antisocial behaviour and can also act as a protective factor moderating the relationship between the risk factor parental mental health problems and antisocial behaviour over time. In view of the fact that antisocial behaviour is a common behavioural problem in childhood and adolescence, causing significant impairments in various areas of life, our results are relevant to clinical practice and should be confirmed by future research. To prevent impairments and long-term consequences, future prevention and intervention programs may benefit from focusing on enhancing social competencies as well as on promoting family functioning and cohesion, particularly in children of parents with a mental disorder.

## Supplementary Information

**Additional file 1: Table S1.** Predicting the initial state and change of antisocial behaviour in 11 to 13 year olds. **Table S2.** Predicting the initial state and change of antisocial behaviour in 14 to 17 year olds. **Table S3.** Resource factors moderating the relationship between parental mental health problems and antisocial behaviour in children and adolescents.

## Data Availability

The datasets generated and analysed during the current study are not publicly available, but are available from the corresponding author on reasonable request.
