# Supplementary material for: Risk and resource factors of antisocial behaviour in children and adolescents: results of the longitudinal BELLA study
Source: Child Adolesc Psychiatry Ment Health. 2021 Oct 22;15:61. doi: 10.1186/s13034-021-00412-3 (PMC8539834; doi:10.1186/s13034-021-00412-3)
Supplement: Supplementary file 1 — Additional file 1: Table S1. Predicting the initial state and change of antisocial behaviour in 11 to 13 year olds. Table S2. Predicting the initial state and change of antisocial behaviour in 14 to 17 year olds. Table S3. Resource factors moderating the relationship between parental mental health problems and antisocial behaviour in children and adolescents. [file 13034_2021_412_MOESM1_ESM.docx]

**Table S1 Predicting the initial state and change of antisocial behaviour in 11- to 13-year olds**

|  | **Regression Model A0^1^**  **predicting**  **initial antisocial behaviour** | | |  | **Regression Model B0^2^**  **predicting**  **change in antisocial behaviour** | | |  |
| --- | --- | --- | --- | --- | --- | --- | --- | --- |
|  | ***b*** | **β** | ***p*** |  | ***b*** | **β** | ***p*** | |
| *Constant* | 0.12 |  | <.001 |  | 0.00 |  | .280 | |
| **Sociodemographic data^3^** |  |  |  |  |  |  |  | |
| Female | -0.01 | -0.03 | .457 |  | 0.00 | -0.03 | .460 | |
| Age 12 years (ref. 11 years) | -0.01 | -0.05 | .225 |  | 0.00 | 0.05 | .294 | |
| Age 13 years (ref. 11 years) | 0.02 | 0.06 | .125 |  | 0.00 | 0.02 | .719 | |
| Socioeconomic status (at baseline) | 0.00 | -0.14 | <.001 |  | 0.00 | 0.13 | .002 | |
| Migration background | 0.00 | 0.00 | .917 |  | 0.00 | -0.03 | .485 | |
| **Familial and parental risks** |  |  |  |  |  |  |  | |
| Not living with both biological parents | 0.02 | 0.06 | .097 |  | 0.00 | 0.03 | .419 | |
| Parental chronic disease (at least one parent) | 0.00 | 0.02 | .640 |  | 0.00 | 0.05 | .228 | |
| Parental strain | 0.06 | 0.24 | <.001 |  | -0.01 | -0.24 | <.001 | |
| **Comorbid mental health problems** |  |  |  |  |  |  |  | |
| Initial symptoms of ADHD (intercept) | 0.12 | 0.39 | <.001 |  | 0.01 | 0.11 | .034 | |
| Change in symptoms of ADHD (slope) |  |  |  |  | 0.05 | 0.17 | <.001 | |
| Initial depressive symptoms (intercept) | 0.06 | 0.07 | .141 |  | -0.01 | -0.05 | .339 | |
| Change in depressive symptoms (slope) |  |  |  |  | 0.20 | 0.12 | .007 | |
| Initial symptoms of generalised anxiety (intercept) | -0.03 | -0.05 | .268 |  | 0.01 | 0.11 | .032 | |
| Change in symptoms of generalised anxiety (slope) |  |  |  |  | 0.00 | -0.01 | .907 | |
| **Risk factor** |  |  |  |  |  |  |  | |
| Initial parental mental health problems (intercept) | 0.02 | 0.05 | .220 |  | 0.01 | 0.08 | .242 | |
| Change in parental mental health problems (slope) |  |  |  |  | 0.02 | 0.06 | .315 | |
| **Resource factors** |  |  |  |  |  |  |  | |
| Initial self-efficacy (intercept) | -0.01 | -0.02 | .542 |  | 0.01 | 0.06 | .249 | |
| Change in self-efficacy (slope) |  |  |  |  | 0.03 | 0.10 | .025 | |
| Initial family climate (intercept) | -0.02 | -0.06 | .161 |  | -0.01 | -0.20 | <.001 | |
| Change in family climate (slope) |  |  |  |  | -0.02 | -0.11 | .017 | |
| Initial social support (intercept) | -0.02 | -0.08 | .049 |  | 0.01 | 0.22 | <.001 | |
| Change in social support (slope) |  |  |  |  | 0.03 | 0.15 | .002 | |

^1^Linear regression Model A0 *(n* = 532); model fit: adjusted *R^2^* = .41; *F* = 25.15; ^2^linear regression Model B0 (*n* = 532); model fit: adjusted *R^2^* = .12; *F* = 4.24; **^3^** we entered all variables simultaneously;

ADHD = Attention-deficit/hyperactivity disorder; *b* = unstandardised regression coefficient; β = standardised regression coefficient; for measures see text (Methods).

**Table S2 Predicting the initial state and change of antisocial behaviour in 14- to 17-year olds**

|  | **Regression Model A0^1^**  **predicting**  **initial antisocial behaviour** | | |  | **Regression Model B0^2^**  **predicting**  **change in antisocial behaviour** | | |  |
| --- | --- | --- | --- | --- | --- | --- | --- | --- |
|  | ***b*** | **β** | ***p*** |  | ***b*** | **β** | ***p*** | |
| *Constant* | 0.13 |  | <.001 |  | 0.01 |  | .034 | |
| **Sociodemographic data^3^** |  |  |  |  |  |  |  | |
| Female | -0.01 | -0.03 | .416 |  | 0.00 | 0.05 | .247 | |
| Age 15 years (ref. 14 years) | 0.02 | 0.05 | .273 |  | -0.01 | -0.08 | .083 | |
| Age 16 years (ref. 14 years) | 0.03 | 0.09 | .038 |  | -0.01 | -0.18 | <.001 | |
| Age 17 years (ref. 14 years) | 0.01 | 0.03 | .531 |  | -0.01 | -0.14 | .004 | |
| Socioeconomic status (at baseline) | 0.00 | -0.10 | .003 |  | 0.00 | -0.05 | .182 | |
| Migration background | 0.00 | 0.00 | .893 |  | -0.01 | -0.06 | .115 | |
| **Familial and parental risks** |  |  |  |  |  |  |  | |
| Not living with both biological parents | 0.04 | 0.09 | .006 |  | 0.00 | -0.01 | .802 | |
| Parental chronic disease (at least one parent) | -0.01 | -0.02 | .543 |  | 0.00 | -0.04 | .327 | |
| Parental strain | 0.06 | 0.23 | <.001 |  | -0.01 | -0.19 | <.001 | |
| **Comorbid mental health problems** |  |  |  |  |  |  |  | |
| Initial symptoms of ADHD (intercept) | 0.16 | 0.39 | <.001 |  | -0.01 | -0.09 | .081 | |
| Change in symptoms of ADHD (slope) |  |  |  |  | 0.03 | 0.09 | .064 | |
| Initial depressive symptoms (intercept) | 0.13 | 0.18 | <.001 |  | 0.00 | 0.01 | .872 | |
| Change in depressive symptoms (slope) |  |  |  |  | -0.17 | -0.09 | .033 | |
| Initial symptoms of generalised anxiety (intercept) | -0.04 | -0.07 | .159 |  | 0.00 | -0.01 | .881 | |
| Change in symptoms of generalised anxiety (slope) |  |  |  |  | -0.02 | -0.05 | .246 | |
| **Risk factor** |  |  |  |  |  |  |  | |
| Initial parental mental health problems (intercept) | -0.02 | -0.06 | .182 |  | 0.01 | 0.10 | .109 | |
| Change in parental mental health problems (slope) |  |  |  |  | 0.01 | 0.02 | .774 | |
| **Resource factors** |  |  |  |  |  |  |  | |
| Initial self-efficacy (intercept) | 0.07 | 0.12 | .003 |  | 0.00 | -0.04 | .448 | |
| Change in self-efficacy (slope) |  |  |  |  | -0.01 | -0.01 | .751 | |
| Initial family climate (intercept) | -0.04 | -0.10 | .016 |  | -0.01 | -0.06 | .201 | |
| Change in family climate (slope) |  |  |  |  | -0.04 | -0.15 | .001 | |
| Initial social support (intercept) | 0.00 | 0.01 | .839 |  | 0.00 | 0.05 | .338 | |
| Change in social support (slope) |  |  |  |  | 0.00 | 0.01 | .830 | |

^1^Linear regression Model A0 *(n* = 613); model fit: adjusted *R^2^* = .32; *F* = 19.24; ^2^linear regression Model B0 (*n* = 613); model fit: adjusted *R^2^* = .08; *F* = 3.31; **^3^** we entered all variables simultaneously;

ADHD = Attention-deficit/hyperactivity disorder; *b* = unstandardised regression coefficient; β = standardised regression coefficient; for measures see text (Methods).

**Table S3 Resource factors moderating the relationship between parental mental health problems and antisocial behaviour in children and adolescents**

|  | **Regression Model A1^1^ predicting**  **initial antisocial behaviour** | | | **Regression Model B1^2^ predicting**  **change in antisocial behaviour** | | |
| --- | --- | --- | --- | --- | --- | --- |
|  | ***b*** | **β** | ***p*** | ***b*** | **β** | ***p*** |
| *Constant* | 0.13 |  | <.001 | 0.00 |  | .631 |
| **Sociodemographic data^3^** |  |  |  |  |  |  |
| Female | -0.01 | -0.03 | 0.295 | 0.00 | 0.02 | .555 |
| Age (in years at baseline) | 0.01 | 0.10 | 0.009 | 0.00 | -0.13 | .006* |
| Age by gender | 0.00 | 0.02 | 0.581 | 0.00 | 0.01 | .837 |
| Socioeconomic status (at baseline) | 0.00 | -0.11 | <.001* | 0.00 | 0.03 | .332 |
| Migration background | 0.00 | 0.00 | 0.960 | 0.00 | -0.04 | .190 |
| **Familial and parental risks^3^** |  |  |  |  |  |  |
| Not living with both biological parents | 0.04 | 0.09 | <.001 | 0.00 | 0.02 | .576 |
| Parental chronic disease (at least one parent) | 0.00 | 0.00 | .879 | 0.00 | -0.01 | .774 |
| Parental strain | 0.06 | 0.23 | <.001* | -0.01 | -0.22 | <.001* |
| **Comorbid mental health problems^3^** |  |  |  |  |  |  |
| Initial symptoms of ADHD (intercept) | 0.14 | 0.39 | <.001* | 0.00 | 0.00 | .935 |
| Change in symptoms of ADHD (slope) |  |  |  | 0.04 | 0.13 | <.001* |
| Initial depressive symptoms (intercept) | 0.11 | 0.14 | <.001* | -0.01 | -0.05 | .194 |
| Change in depressive symptoms (slope) |  |  |  | 0.00 | 0.00 | .950 |
| Initial symptoms of generalised anxiety (intercept) | -0.04 | -0.07 | .033 | 0.01 | 0.06 | .158 |
| Change in symptoms of generalised anxiety (slope) |  |  |  | -0.01 | -0.02 | .463 |
| **Risk factor^3^** |  |  |  |  |  |  |
| Initial parental mental health (intercept) | -0.01 | -0.02 | .543 | 0.01 | 0.08 | .085 |
| Change in parental mental health (slope) |  |  |  | 0.01 | 0.02 | .648 |
| **Resource factors^3^** |  |  |  |  |  |  |
| Initial self-efficacy (intercept) | 0.03 | 0.06 | .042 | 0.00 | 0.00 | .931 |
| Change in self-efficacy (slope) |  |  |  | 0.01 | 0.04 | .212 |
| Initial family climate (intercept) | -0.03 | -0.09 | .002 | -0.01 | -0.12 | .001* |
| Change in family climate (slope) |  |  |  | -0.03 | -0.12 | <.001* |
| Initial social support (intercept) | -0.01 | -0.02 | .539 | 0.01 | 0.10 | .006* |
| Change in social support (slope) |  |  |  | 0.01 | 0.05 | .124 |
| **Interactions between risk and resource factors^3^** |  |  |  |  |  |  |
| Initial parental mental health problems by initial self-efficacy | 0.02 | 0.01 | .660 | -0.01 | -0.04 | .285 |
| Initial parental mental health problems by change in self-efficacy |  |  |  | -0.03 | -0.04 | .251 |
| Change in parental mental health problems by initial self-efficacy |  |  |  | -0.03 | -0.02 | .627 |
| Change in parental mental health problems by change in self-efficacy |  |  |  | -0.15 | -0.04 | .266 |
| Initial parental mental health problems by initial family climate | 0.01 | 0.01 | .794 | -0.01 | -0.08 | .098 |
| Initial parental mental health problems by change in family climate |  |  |  | -0.03 | -0.04 | .294 |
| Change in parental mental health problems by initial family climate |  |  |  | -0.04 | -0.05 | .269 |
| Change in parental mental health problems by change in family climate |  |  |  | -0.24 | -0.10 | .020 |
| Initial parental mental health problems by initial social support | -0.02 | -0.03 | .353 | 0.00 | 0.00 | .970 |
| Initial parental mental health problems by change in social support |  |  |  | 0.02 | 0.03 | .411 |
| Change in parental mental health problems by initial social support |  |  |  | -0.02 | -0.03 | .522 |
| Change in parental mental health problems by change in social support |  |  |  | 0.09 | 0.03 | .352 |

^1^Linear regression Model A1 *(n* = 1,145); model fit: adjusted *R^2^* = .35; *F* = 35.30; ^2^linear regression Model B1 (*n* = 1,145); model fit: adjusted *R^2^* = .07; *F* = 3.47; **^3^** all variables were entered simultaneously.

ADHD = Attention-deficit/hyperactivity disorder; *b* = unstandardised regression coefficient; β = standardised regression coefficient; for measures see text (Methods).
